# ESR Essentials: imaging in nasal obstruction and epistaxis—practice recommendations by the European Society of Head and Neck Radiology

**DOI:** 10.1007/s00330-025-12305-6

**Published:** 2026-02-16

**Authors:** Anne R. J. Péporté, Edith Vassallo, Lorenzo Preda, Timothy Beale, Jussi Hirvonen

**Affiliations:** 1 Department of Radiology, Cantonal Hospital Frauenfeld, Frauenfeld, Switzerland; 2 Institute of Diagnostic and Interventional Radiology, University Zurich, Zurich, Switzerland; 3 Department of Medical Imaging, Mater Dei Hospital, Msida Malta, Malta; 4 Department of Clinical-Surgical, Diagnostic and Pediatric Sciences, University of Pavia, Pavia, Italy; 5 Radiology Institute, Fondazione IRCCS Policlinico San Matteo, Pavia, Italy; 6 Department of Radiology, University College London Hospitals, London, United Kingdom; 7 Department of Radiology, University of Turku and Turku University Hospital, Turku, Finland; 8 Department of Radiology, Faculty of Medicine and Health Technology and Tampere University Hospital, Tampere University, Tampere, Finland

**Keywords:** Nasal obstruction, Epistaxis, Computed tomography, Magnetic resonance imaging, Digital subtraction angiography

## Abstract

**Abstract:**

Nasal obstruction and epistaxis are common otorhinolaryngologic complaints with multiple etiologies ranging from benign anatomical variations and mucosal inflammation to severe neoplastic and vascular disorders. Clinical evaluation and nasal endoscopy are first-line diagnostic tools, with imaging reserved for selected indications. Imaging differentiates uncomplicated from complicated presentations, guiding management and surgical planning. Computed tomography (CT) is the primary modality for assessing sinonasal anatomical variants, bony pathology, and inflammatory or neoplastic disease. Magnetic resonance imaging (MRI) complements CT when soft tissue or intracranial extension is suspected. Imaging in epistaxis is not routinely indicated but is recommended in recurrent, severe, or posterior bleeding to detect underlying vascular lesions or tumors. CT angiography is preferred for vascular assessment and interventional planning. Implementing these recommendations can improve diagnostic accuracy, streamline patient management, and enhance surgical outcomes in patients presenting with nasal obstruction and epistaxis.

**Key Points:**

- *Clinical history and nasal endoscopy should be the primary tools for initial assessment, with imaging reserved for inconclusive or persistent cases.*
- *CT is the preferred imaging modality for evaluating structural causes of nasal obstruction, especially prior to surgical intervention.*
- *Imaging in epistaxis is indicated mainly in severe, recurrent, or posterior bleeding to localize the source and guide treatment.*

## Key recommendations

- Initial clinical assessment and nasal endoscopy should guide the need for imaging in patients presenting with nasal obstruction or epistaxis. Level of Evidence: 2a/Class of Recommendation: I (Strongly recommended).
- Non-contrast CT is the preferred imaging modality for evaluating structural abnormalities in nasal obstruction and is recommended prior to surgical intervention. Level of Evidence: 1b/Class of Recommendation: I (Strongly recommended).
- Imaging in epistaxis should be reserved for patients with severe, recurrent, or posterior bleeding to localize vascular sources and rule out underlying pathology. Level of Evidence: 2b/Class of Recommendation: IIa (Reasonable to perform).

## Introduction

Nasal obstruction and epistaxis (nosebleeds) frequently present in clinical practice, affecting all age groups and often caused by benign, self-limiting conditions. However, certain cases indicate serious and potentially life-threatening underlying conditions that need prompt diagnosis and treatment.

The initial assessment for both nasal obstruction and epistaxis relies heavily on clinical history and endoscopic nasal examination. Imaging is pivotal when clinical findings are inconclusive, the clinical presentation is complicated, or when surgical or endovascular intervention is planned.

This article provides practice recommendations for imaging choices and diagnostic considerations when evaluating nasal obstruction and epistaxis, thereby integrating international evidence-based guidelines to inform best practice approaches suitable for general radiologists.

## Background

### Nasal obstruction

#### Epidemiology and etiology

Nasal obstruction (NO) is the sensation of reduced airflow or fullness in the nostrils that causes difficulty breathing through the nose, often impairing sleep and quality of life. It is commonly reported in primary care, otorhinolaryngology, and allergy clinics. The condition is estimated to affect approximately 30–40% of the general population, though precise data are lacking [1].

**Fig. 1:**
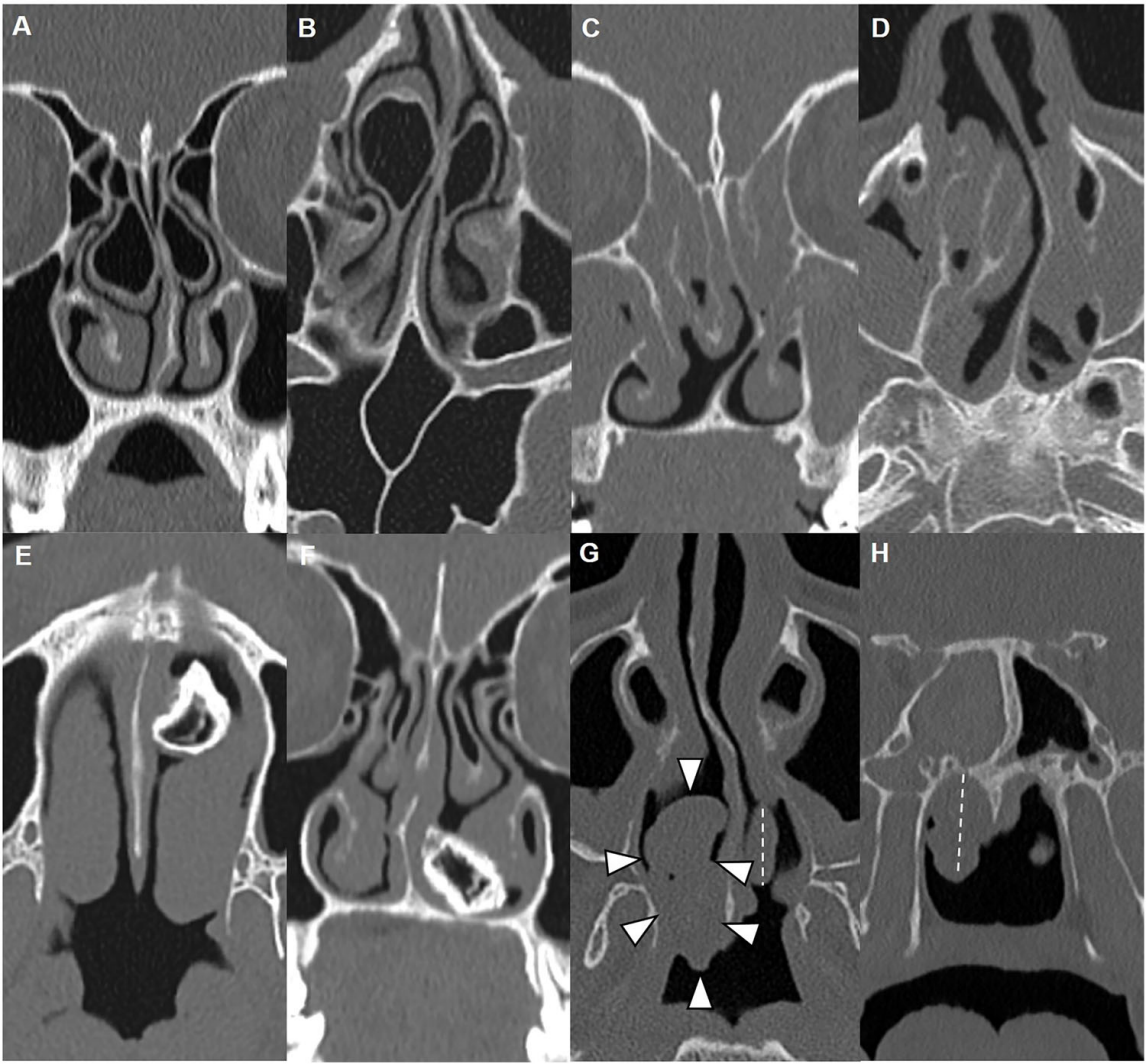
Overview of benign structural causes of nasal obstruction and epistaxis. Coronal (**A**, **C**, **F**, **H**) and axial (**B**, **D**, **E**, **G**) bone window sinonasal CT images. There is an enlarged and pneumatized middle turbinate on both sides with narrowing of the ostiomeatal complex (**A**, **B**). Left-convex nasal septal deviation (**C**, **D**) with architectural distortion and opacification of the ostiomeatal complex on both sides. There is also opacification of the maxillary sinus and the ethmoid air cells on both sides. A well-defined, rounded calcified lesion, consistent with a rhinolith (**E**, **F**), is noted between the left inferior meatus and nasal septum. There is a deviation of the nasal septum to the right. No evidence of bony destruction. Mucosal thickening also noted in the nasal fossa. Well-circumscribed nasal polyps (**G**, **H**) partially obliterating the posterior aspect of the right nasal cavity and protruding into the nasopharynx (white triangles in **G**) and a smaller lesion on the contralateral nasal cavity (dashed line in **G**). There are also findings of chronic sphenoid sinusitis on the right

Nasal obstruction is most often due to mucosal (physiological) causes, with structural, neoplastic, and iatrogenic factors being less common. The commonest causes of nasal obstruction are rhinitis and chronic rhinosinusitis. Systemic conditions such as GPA, sarcoidosis, and cocaine use can cause nasal obstruction. Turbinate enlargement (Fig. 1A, B) and septal deviation (Fig. 1C, D) are common structural causes of nasal obstruction and frequent indications for surgery (e.g., septoplasty). Nasal valve dysfunction can significantly impair nasal breathing, particularly in the absence of other obstructive lesions. It is frequently associated with secondary mucosal changes due to altered airflow dynamics [2].

**Fig. 2:**
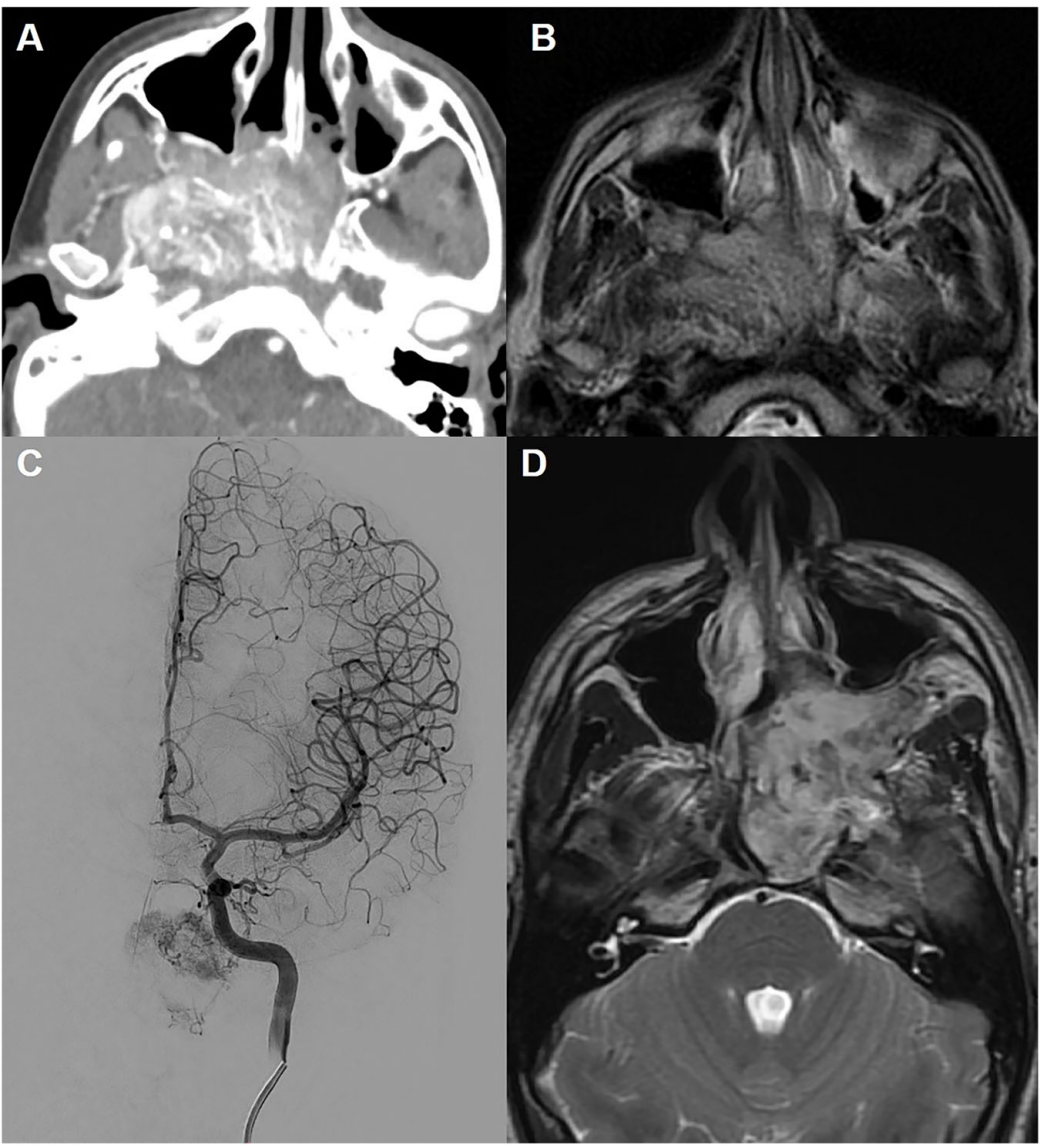
Juvenile nasopharyngeal angiofibroma (JNA). Axial CT angiogram (**A**), axial T2w MR image (**B**). A large, highly vascular soft tissue lesion is noted in the posterior choana and nasopharynx, compromising the nasopharyngeal airway. It extends to the left expanded and remodeled pterygopalatine fossa. From the pterygopalatine fossa, it extends through the pterygomaxillary fissure to the right infratemporal fossa and masticator space. Another JNA case: Digital subtraction angiogram (**C**) and axial T2w MR image (**D**). There is a sizeable arterial blush corresponding to the known left-sided JNA. There are arterial feeders arising from the ipsilateral internal carotid artery, the internal maxillary artery and the left ophthalmic artery. On MR, there is an inhomogeneous, well-circumscribed mass centered on the widened left sphenopalatine foramen with extension into the nasal cavity and nasopharynx. There is also widening of the left pterygomaxillary fissure with a little extension into the infratemporal fossa and masticator space

Sinonasal neoplasms are rare, accounting for only 3% of head and neck cancers [3], with malignant lesions being more common and generally carrying a poor prognosis. Malignancies such as squamous cell carcinoma (80% of cases) and other variants often present with nonspecific symptoms like nasal obstruction, pain, or epistaxis, and can invade adjacent structures, causing severe complications; risk factors include smoking, chemical exposure, and HPV infection [4]. Among benign tumors, inverted papilloma requires radical surgery due to malignant potential [5], while juvenile nasopharyngeal angiofibroma (Fig. 2), typically in adolescent males, has characteristic imaging features reflecting its vascular and locally invasive growth pattern [6].

### Epistaxis

#### Epidemiology and etiology

Epistaxis (nosebleed) is a frequent emergency in otolaryngology departments across Europe. The incidence of epistaxis visits to emergency departments (ED) has been reported as approximately 1 in 30 ED visits, serving a population coverage of about 77 per 100,000 people.

**Fig. 3:**
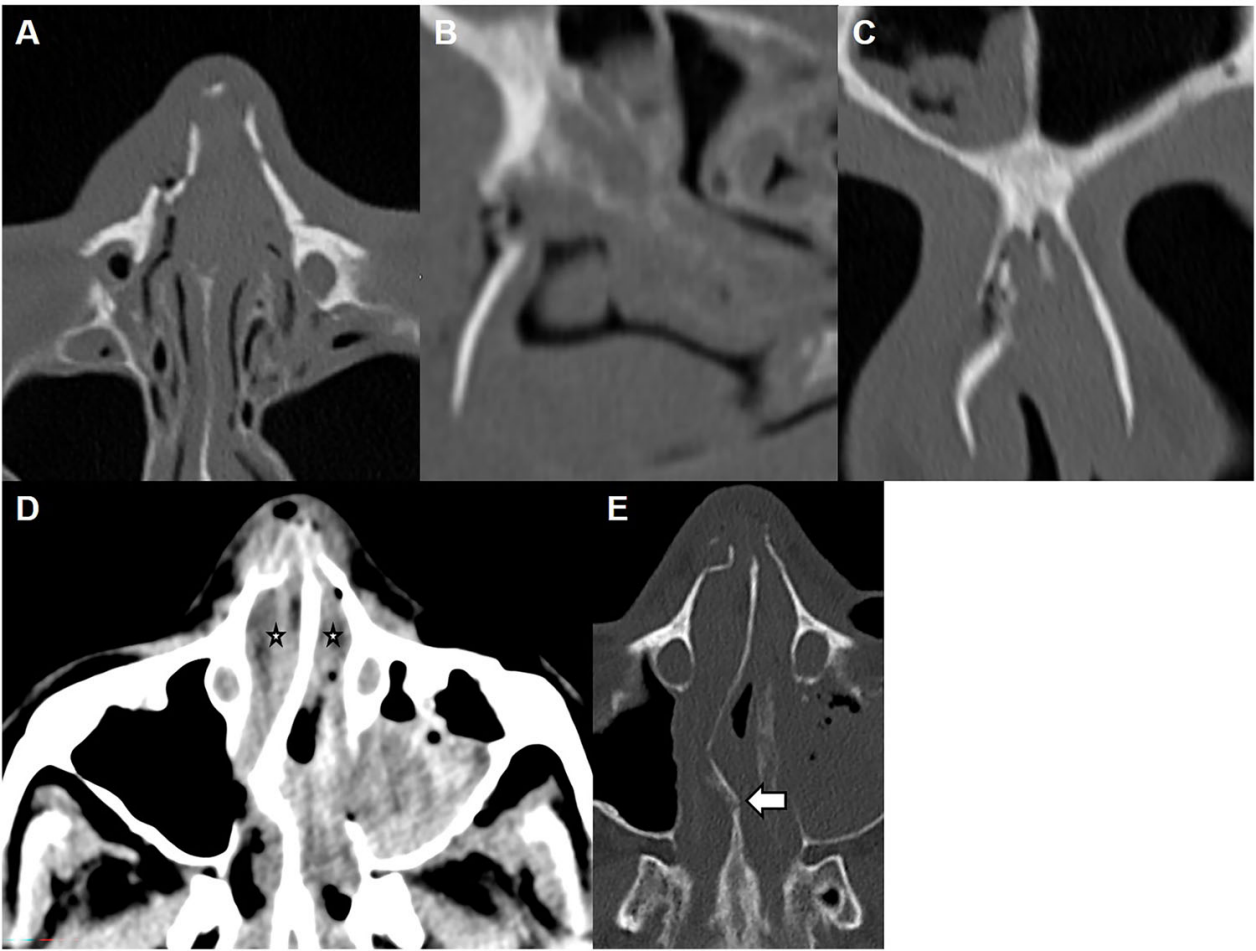
Facial fractures. Axial (**A**, **E**), sagittal (**B**) and coronal (**C**) bone window and axial soft tissue window (**D**) CT images of the nasal bone and septum revealing a dislocated fracture of the right nasal bone (**A**–**C**). The lower images show a displaced nasal bone and septum fracture (**E**) as well as a bilateral nasal septal hematoma (star in **D**)

Trauma is an important cause of epistaxis in an otherwise healthy population, including accidental injury, facial fractures (Fig. 3), or nasal mucosal laceration [7]. Iatrogenic causes, including surgical complications, endoscopy, or nasal packing, are common in hospital and procedural settings [8].

Anterior epistaxis, the most common type in children and young adults, usually arises from Kiesselbach’s plexus and responds well to conservative measures or nasal packing [9]. Posterior epistaxis, less common (5–10%) and typically originating from the Woodruff plexus in older patients, is harder to control [10]. Systemic factors are frequent: nearly half of hospitalized cases involve comorbidities, with hypertension, anticoagulant/antiplatelet use, and coagulation disorders being notable contributors [11]. Epistaxis is also linked to inherited bleeding disorders (e.g., von Willebrand disease, hemophilia) [12] and vascular abnormalities (e.g., hereditary hemorrhagic telangiectasia), while newer anticoagulants have raised concerns and guided recent management guidelines [13].

### Imaging modalities for nasal obstruction and epistaxis

#### Conventional radiography (CR)

CR is rarely used for nasal obstruction or epistaxis due to poor accuracy and has been replaced by CT and MRI, which offer superior bone and soft tissue detail.

#### Ultrasound (US)

US has a limited but valuable role in evaluating superficial nasal and perinasal soft tissue lesions. The bony framework of the nasal cavity and sinuses limits imaging clarity of intranasal structures, reducing its effectiveness in diagnosing nasal obstruction or epistaxis causes. While not a standard modality for routine evaluation, US offers a quick, non-invasive, radiation-free option in select cases—particularly for guiding aspiration/biopsy or in pediatric and pregnant patients where radiation avoidance is preferred [14].

#### Computed tomography (CT), CT angiography and PET/CT

CT plays a central role in the evaluation of nasal obstruction and epistaxis, with an important distinction between non-contrast and contrast-enhanced techniques.

Non-contrast CT (NCCT) of the sinuses is the first-line imaging modality for evaluating persistent or complicated nasal obstruction. It provides excellent visualization of the bony anatomy and paranasal sinuses, allowing detailed assessment of sinonasal masses, sinus opacification, mucosal disease, bone remodeling, erosion, or destruction. Thin-slice NCCT is recommended by the ACR Appropriateness Criteria in patients suspected of having nasal polyps, granulomatous disease, neoplasms, or traumatic injury [14]. NCCT offers precise mapping of lesions, aiding surgical planning and differentiating inflammatory from neoplastic processes. CT is indicated when there is inadequate response to medical therapy for mucosal disease or when “red flag” features are present, such as persistent unilateral obstruction, epistaxis, pain, or orbital and neurological symptoms. Additional indications include the presence of a mass detected on endoscopy in the nasal cavity or nasopharynx, as well as associated lymphadenopathy.

Contrast-enhanced CT (CECT), on the other hand, is not routinely used for bony and mucosal evaluation but may be helpful in specific contexts such as better delineation of soft tissue masses or suspected vascular lesions. PET-CT is used for staging sinonasal malignancies and detecting metastases [15].

CT Angiography (CTA) is particularly useful for diagnosing epistaxis when bleeding is posterior, recurrent, or severe. CTA is a contrast-enhanced study that enables non-invasive vascular mapping to localize the bleeding source, identify vascular malformations, pseudoaneurysms, or tumors with a rich blood supply, and assists in planning endovascular interventions such as embolization. According to the ACR Appropriateness Criteria, CTA is appropriate when initial conservative management fails or when invasive therapy is considered, as it provides rapid, high-resolution images of vascular and adjacent anatomical structures [14].

An emerging technique, dual-energy CT (DECT), may enhance the characterization of soft tissue tumors [16] or differentiate between active bleeding or vascular lesions from retained blood or sinus secretions, potentially improving diagnostic confidence.

Cone Beam CT offers high-resolution 3D imaging of sinonasal bony structures with lower radiation than conventional CT. It is useful for detailed anatomical assessment but has limited soft tissue contrast and is often complementary to standard CT [3, 14].

For recommended diagnostic reference levels (DRLs) for CT imaging of the sinonasal region, see Table 1.

**Table 1:** Technical parameters for sinonasal and dual-energy CT imaging

Parameter	Reference value	Unit	Notes
CTDI vol	4–10	mGy	Volume CT dose index for sinonasal CT
Dose length product (DLP)	100–250	mGy.cm	Total dose measure for the complete exam
Tube voltage	~100	kV	Low kV protocols optimize dose
Reconstruction technique	Iterative reconstruction (IR), deep learning-based (AiCE)		Dose reduction and image quality enhancement
Reconstruction planes	Axial, sagittal, coronal		Multiple planes for 3D assessment
Slice thickness	0.25–3	mm	Thicker reconstruction slices (2–3 mm) are suggested for optimal soft tissue evaluation to enhance contrast resolution and reduce noise, while thinner slices (< 1 mm) improve spatial resolution for detailed anatomical assessment
Kernel	Bone kernel and Body/soft tissue kernel		Depending on the tissue contrast required
Dual-energy CT	e.g., 80/140 kVp or similar combinations	kVp	Dual-energy protocols use mixed kVp for tissue differentiation
Contrast media	1–2 mL/kg iodine; 3–5 mL/s rate	mL/kg, mL/s	Timing: arterial 20–30 s, venous 60–70 s. Dual-energy CT allows dose optimization and tailored delay for the best contrast enhancement.

This table summarizes the key technical parameters recommended for sinonasal CT imaging, including dose metrics (CTDI Vol, DLP), tube voltage, slice thickness, reconstruction planes, kernel types, and iterative reconstruction techniques. Dual-energy CT protocols are also summarized with typical combinations of tube voltages used. These parameters aim to optimize image quality while minimizing radiation dose

#### Magnetic resonance imaging (MRI)

The intrinsic tissue contrast of MRI, with or without a gadolinium-based contrast agent, can be used to assess the soft tissue components of a mass. Although the imaging appearance of soft tissue masses can be nonspecific, MRI occasionally reveals imaging patterns indicative of specific pathologies. MRI can show the characteristic convoluted cerebriform pattern of IP on T2-weighted and contrast-enhanced T1-weighted images, the intrinsic T1 hyperintensity of melanotic melanomas, and peritumoral intracranial cysts that suggest esthesioneuroblastoma [17].

Quantitative MRI parameters such as lower T2 signal intensity [18] and lower apparent diffusion coefficient (ADC) values on diffusion-weighted imaging (DWI) correspond with higher tumor cellularity and may suggest malignancy [19].

Perfusion MRI, obtained through dynamic contrast-enhanced (DCE) sequences, can provide additional functional information about tumor vascularity and aggressiveness. However, its clinical application is limited by technical complexity and moderate diagnostic accuracy.

MRI is superior to CT in tumor mapping due to better differentiation of tumor tissue from T2 hyperintense sinus inflammation and retained secretions. MRI also excels at detecting intraorbital extension, intracranial spread, perineural invasion, and osseous marrow infiltration—all critical for accurate tumor staging and surgical planning.

At least one sequence should be acquired in each plane (axial, coronal, sagittal) or alternatively a volume sequence, with particular attention to sagittal images after anterior skull base reconstruction. Slice thickness ~2–3 mm is optimal for balancing spatial resolution and signal-to-noise ratio. Fat-suppressed sequences enhance the delineation of tumor from fat and inflammation (see Table 2 for detailed sinonasal MR protocol recommendation).

**Table 2:** Recommended MRI protocol for sinonasal tumor imaging

Sequence	Purpose	Technical details and comments
T1-weighted (SE or FSE)	Anatomical detail, marrow assessment	Axial, coronal planes; slice thickness ~3 mm
T2-weighted (TSE or FSE)	Fluid-sensitive, soft tissue contrast	Axial, coronal, sagittal planes; fat suppression optional
Contrast-enhanced T1-weighted	Enhance lesion characterization	Fat-saturated, high-resolution; axial and coronal planes
Diffusion-weighted Imaging (DWI)	Assess cellularity, differentiate tumor vs edema	b-values 500 and 1000 s/mm²; ADC maps generated
Dynamic contrast-enhanced (DCE) MRI	Functional perfusion analysis	T1-weighted gradient echo; temporal resolution 4–8 s
STIR or fat-saturation sequences	Highlight edema, marrow involvement	Useful for detecting lymph nodes and bone marrow edema
3D gradient echo (3D-GE) sequences	High spatial resolution and tissue contrast	Isotropic voxel size ~0.5–0.8 mm, facilitates multiplanar reformatting

This table outlines the suggested MRI sequences for optimal evaluation of sinonasal tumors, detailing their primary diagnostic purpose and key technical parameters. The protocol emphasizes multiplanar acquisition with high‑resolution sequences to assess tumor extent, differentiate neoplastic from inflammatory tissue, and evaluate potential intraorbital, intracranial, perineural, and bone marrow involvement. Optional advanced sequences (DWI, DCE MRI) may provide additional functional or cellularity information, though their use can be limited by availability and diagnostic accuracy

*SE* spin echo, FSE fast spin echo, *TSE* turbo spin echo, *DWI* diffusion‑weighted imaging, *ADC* apparent diffusion coefficient, *DCE* dynamic contrast‑enhanced, *STIR* short tau inversion recovery, *GE* gradient echo, *NCCT* non‑contrast computed tomography, *CTA* CT angiography, *MRI* magnetic resonance imaging

Overall, CT and MRI serve as complementary imaging tools in evaluating sinonasal masses, helping to localize, characterize, and define lesion extent for optimal treatment planning [20, 21].

#### Digital subtraction angiography (DSA) with endovascular embolization

According to the ACR Appropriateness Criteria for sinonasal disease, craniofacial DSA is not useful in the initial imaging evaluation of nasal obstruction from a sinonasal mass. DSA may be useful for preoperative planning, such as characterization of tumor vasculature and preoperative embolization of a vascular mass [14].

While most cases of epistaxis resolve with conservative treatment, about 6% of patients need more invasive interventions beyond cautery or packing due to recurrent or difficult-to-control bleeding [22]. Current treatment protocols now adopt surgical arterial ligation and endovascular embolization as second-line therapies for recurrent or intractable epistaxis.

**Fig. 4:**
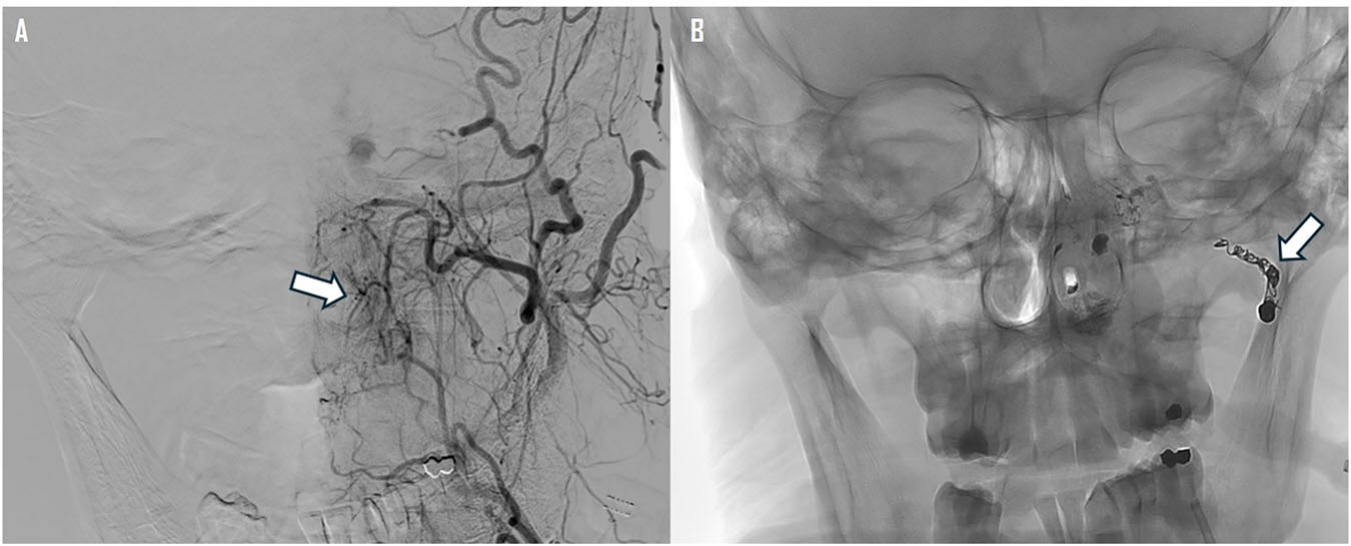
Angiographic images of epistaxis embolization. 47-year-old male patient status post renal transplant. Had left-sided epistaxis on day 1 post-op. Front view angiographic image with subtraction (**A**) revealing a contrast blush from the left maxillary artery. Dense material in the embolized area (**B**) corresponds to the radiopaque component of the tamponade. The primary embolization was performed using liquid embolic agents, specifically NBCA (n-butyl-cyanoacrylate) combined with lipiodol, with coiling as a second-line measure to augment occlusion of the maxillary artery. Front view conventional radiographic image confirming complete occlusion of the left maxillary artery after deploying Tornado coils (4–6 mm) into the feeding vessels

DSA with embolization is mainly used for posterior epistaxis, targeting bilateral sphenopalatine and distal internal maxillary arteries (Fig. 4), and sometimes facial arteries due to their anastomoses. This approach achieves about an 87% control rate, with minor complications in 20% of cases and major ones, including necrosis, paralysis, blindness, or stroke, occurring in 2.1–3.8% of cases [23].

### Key findings and differential diagnosis

#### Structural abnormalities

These include nasal septal deviation, turbinate abnormalities and anatomical variants such as concha bullosa or Haller and Onodi cells. Nasal valve obstruction involves structural or dynamic compromise of the internal or external nasal valve, which are critical narrow segments regulating airflow resistance. Causes include congenital narrow valve angles, trauma, previous surgery, or collapse of the lateral nasal wall during inspiration. Non‑contrast CT of the paranasal sinuses allows precise assessment of bony landmarks, the nasal valve area, and spatial relationships critical for surgical planning [24].

Beyond causing obstruction, these abnormalities can predispose to epistaxis. Septal deviation and turbinate hypertrophy may increase mucosal drying and fragility through turbulent airflow, leading to recurrent anterior bleeding. Studies report a higher prevalence of septal deviation or turbinate hypertrophy among patients with epistaxis, with bleeding often occurring on the deviated side. Thus, altered airflow and mucosal irritation are shared mechanisms linking these abnormalities to both obstruction and nosebleeds [24, 25].

#### Mucosal conditions

Mucosal disorders include allergic and non‑allergic rhinitis as well as chronic rhinosinusitis. Imaging reveals inflammatory mucosal thickening, polypoid changes, and sinus opacification. Granulomatous diseases such as sarcoidosis, granulomatosis with polyangiitis, and fungal sinusitis can show characteristic features, including calcifications or bone erosion/remodeling.

These conditions increase the risk of epistaxis by causing chronic inflammation, edema, and mucosal friability, making the nasal lining prone to bleeding—particularly in the anterior nasal cavity, where the vascular network is dense [2].

#### Trauma and previous surgery

Nasal and mid‑facial fractures and postsurgical changes such as scarring or mucosal synechiae can alter nasal airflow and sinus drainage. Prior surgical changes—such as widened ostiomeatal complexes, opened sinus walls, or graft material—must be distinguished radiologically from residual or recurrent disease and correlated with operative history.

Trauma and surgery are well‑recognized causes of epistaxis, either immediately through vessel injury or later via mucosal ulceration or septal perforation [26].

#### Neoplastic processes (benign and malignant)

**Fig. 5:**
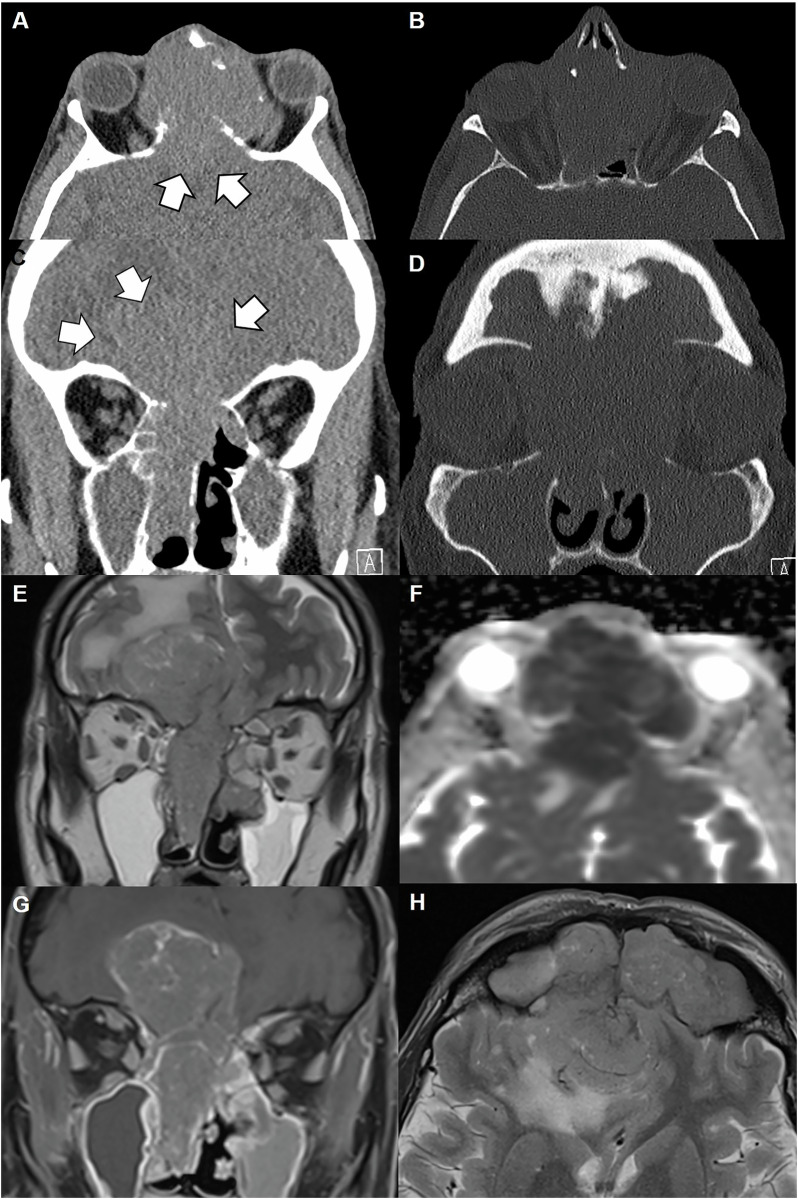
Undifferentiated sinonasal squamous cell carcinoma (SCC). Axial (**A**) and coronal (**C**) soft tissue window and axial (**B**) and coronal (**D**) bone window CT images. Coronal T2w (**E**), axial ADC (**F**), coronal T1w post contrast (**G**) and axial T2w (**H**) MR images. There is a large inhomogeneous mass centered in the right nasal cavity with extension through the destructed ethmoid air cells and the lateral orbital walls into the orbits. There is extension through the destructed cribriform plate/planum sphenoidale into the anterior cranial fossa, giving the lesion a “dumbbell” or “waist” like appearance. The very low ADC values indicate high cellularity. There is vasogenic edema of the adjacent frontal lobe

Benign lesions include inverted papillomas and juvenile nasopharyngeal angiofibromas (JNA); most important malignant tumors encompass squamous cell carcinoma (Fig. 5), adenocarcinoma, esthesioneuroblastoma, and sinonasal undifferentiated carcinoma (SNUC). CT is optimal for assessing bone remodeling or destruction, ossification, and calcification, whereas MRI excels at defining soft tissue extent, distinguishing tumor from retained secretions, and detecting perineural, intraorbital, or intracranial spread.

Neoplasms may cause nasal obstruction through space occupation and epistaxis through mucosal ulceration, tumor necrosis, or rich vascularity. Tumor-related epistaxis must be considered in cases of unilateral, recurrent, or severe bleeding. Benign and malignant sinonasal neoplasms and vascular malformations can present with persistent or posterior epistaxis and may require advanced imaging and intervention. Persistent or recurring nosebleeds in teenage boys—especially if the bleeding is only from one nostril and accompanied by nasal obstruction—may indicate JNA, a rare, benign but locally aggressive vascular tumor (Fig. 2C) [6]. Persistent, severe, or recurrent bleeding in this context warrants targeted cross‑sectional imaging to characterize the lesion, determine the extent, and guide biopsy or treatment [27–29].

Regarding imaging after treatment failure, especially when epistaxis recurs or persists, advanced imaging should be performed promptly once clinical assessment indicates suspicion (e.g., within 24–48 h). However, routine imaging for surveillance is generally recommended at 3–4 months post-treatment completion to establish a baseline for recurrence monitoring. MRI is advised every 6 months for the first 3 years in locally advanced tumors (T2-T4) [29]. Early imaging within 24–48 h after treatment failure is typically reserved for emergent management rather than routine evaluation due to the risk of false positives related to post-therapeutic inflammation. PET-CT imaging is optimally timed at 3–6 months post-treatment to reduce false positives and improve specificity for the detection of residual or recurrent disease [1–3]. This imaging strategy, combined with clinical and endoscopic surveillance, is critical for the timely identification of recurrences and guiding further management.

### Uncomplicated versus complicated nasal obstruction and epistaxis

Nasal obstruction and epistaxis can be uncomplicated or complicated, with different implications for imaging. Uncomplicated nasal obstruction, without masses or infection, responds to conservative treatment and is managed clinically or with endoscopy. Complicated cases—persistent, progressive, or involving masses, granulomatous disease, infection, or trauma—require NECT of the sinuses, with MRI added if soft tissue masses are suspected or for surgical planning. Uncomplicated epistaxis, an isolated anterior bleed, is managed conservatively with rhinoscopy or endoscopy, while complicated epistaxis—recurrent, posterior, severe, or linked to tumors, vascular malformations, trauma, or coagulopathy—necessitates targeted imaging, primarily CT angiography, with MRI reserved for suspected neoplasms or detailed intervention planning (see Tables 3, 4).

**Table 3:** Key findings and imaging recommendations for uncomplicated and complicated nasal obstruction and epistaxis

Condition	Key findings—uncomplicated	Recommended imaging—uncomplicated	Key findings—complicated	Recommended imaging—complicated
Nasal obstruction	- No evidence of mass lesions, infection, or structural compromise. - Responds to conservative management.	- Imaging usually not required. - Clinical and/or endoscopic follow-up recommended.	- Persistent or progressive despite treatment. - Suspicion/presence of mass lesions, polyps (Fig. 1G, H), granulomatous disease, neoplasm, complicated infection, or trauma.	- NCCT of paranasal sinuses (first-line). - MRI if soft tissue mass suspected. - Imaging for preoperative planning.
Epistaxis	- Isolated anterior bleeding without underlying pathology.	- No imaging typically needed (per AAO guideline). - Anterior rhinoscopy/endoscopy for localization.	- Recurrent, posterior, or severe bleeding needing intervention. - Suspicion of neoplasm, vascular malformation, trauma, or coagulopathy.	- CTA to localize bleeding source and assess vasculature. - MRI if tumor suspected. - Imaging for interventional or surgical planning.

Summary of the distinguishing clinical features of uncomplicated versus complicated nasal obstruction and epistaxis, along with the corresponding imaging recommendations for each category. “Uncomplicated” cases are typically managed conservatively without imaging, while “complicated” cases often require advanced imaging—most commonly non‑contrast CT for nasal obstruction and CT angiography for epistaxis—to guide diagnosis and treatment planning

**Table 4:** Imaging modalities not recommended for routine use in uncomplicated nasal obstruction and epistaxis

Imaging modality	Reason for avoidance
Plain radiography (X-ray)	Limited sensitivity and specificity; cannot distinguish viral from bacterial infections; not recommended for routine use in uncomplicated cases.
PET/CT	High radiation dose; reserved for oncologic staging; not indicated for uncomplicated nasal obstruction.
MRI	Limited bony detail; not first-line for uncomplicated obstruction; reserved for soft tissue or intracranial extension suspicion.
CT (non-contrast)	Recommended only for complicated cases, surgical planning, or when endoscopic findings are inconclusive.
Ultrasound	Limited use due to poor visualization of sinonasal structures; mainly for superficial soft tissue lesions.

This table summarizes imaging modalities that are not recommended for routine use in uncomplicated nasal obstruction and epistaxis. These recommendations are based on current evidence indicating limited diagnostic value, unnecessary radiation exposure, or insufficient impact on clinical management. The table aims to guide clinicians in selecting appropriate imaging techniques to optimize patient safety and resource utilization. Modalities such as plain radiography and PET/CT should be reserved for specific indications and avoided in uncomplicated cases. CT and MRI use should be limited to when clinically justified by complexity or suspicion of neoplastic or vascular pathology

## Summary statement

**Fig. 6:**
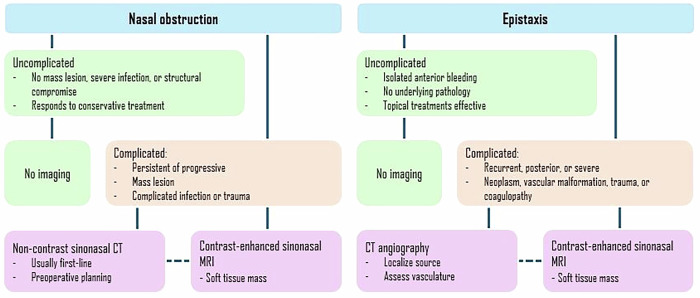
Flowchart for imaging nasal obstruction and epistaxis. Solid lines denote the suggested pathway, and dashed lines denote optional approaches

Nasal obstruction and epistaxis are common clinical presentations, the majority of which are mild in nature and can be adequately assessed through physical examination and nasal endoscopy. Advanced imaging modalities such as CT or MRI are generally indicated only in cases of persistent symptoms, inconclusive endoscopic findings, or when preoperative planning is required. In the context of epistaxis, radiological imaging is reserved for instances where bleeding is severe, recurrent, or suspected to originate from posterior or less accessible sites. These imaging techniques provide detailed visualization of the nasal cavity and vascular structures, thereby facilitating accurate diagnosis and guiding optimal management (Fig. 6). This targeted approach ensures that diagnostic imaging is utilized judiciously, minimizing unnecessary radiation exposure while supporting high-quality patient care.

## Patient summary

Stuffy nose and nosebleeds are common medical problems. Most cases are mild and can be diagnosed through a clinical exam and nasal endoscopy. Imaging tests such as CT or MRI are generally reserved for persistent symptoms, unclear findings on endoscopy, or surgical planning. For nosebleeds, scans are only needed if bleeding is severe, frequent, or comes from deeper areas of the nose. This targeted use of imaging helps doctors accurately identify the cause and choose the best treatment while avoiding unnecessary tests and radiation exposure.
